# Influence of Patient Gender on In‐Hospital Mortality: A Population‐Based Cross‐Sectional Study

**DOI:** 10.1002/nop2.70132

**Published:** 2025-01-10

**Authors:** Nahikari Vizuete‐Aldave, Maider Ugartemendia‐Yerobi, Beatriz Pereda‐Goikoetxea, Nagore Zinkunegi‐Zubizarreta, Josune Zubeldia‐Etxeberria, Udane Elordi‐Guenaga, Haritz Arrieta, Ainitze Labaka

**Affiliations:** ^1^ Department of Nursing II, Faculty of Medicine and Nursing University of the Basque Country (UPV/EHU) Donostia‐San Sebastián Gipuzkoa Spain; ^2^ Biogipuzkoa Health Research Institute Donostia‐San Sebastián Gipuzkoa Spain

## Abstract

**Aim:**

To analyse the association between gender and in‐hospital mortality odds ratios among patients in the Basque Country.

**Design:**

Cross‐sectional study.

**Methods:**

Admission data pertaining to the period between 1 January 2016 and 31 December 2018 were gathered for all registered acute care hospitals (both public and private) in the Basque Country. Odds ratios were calculated through binomial logistic regressions to determine the association between gender and mortality in each diagnostic category of the ICD‐10.

**Results:**

Women had a higher in‐hospital mortality odds ratio for diseases of the circulatory system (OR 1.07 [1.01–1.14], *p* < 0.05). In contrast, men were at greater risk of in‐hospital death from neoplasms (OR 0.86 [0.83–0.94], *p* < 0.05), diseases of the nervous system (OR 0.83 [0.70–0.97], *p* < 0.05), diseases of the genitourinary system (OR 0.83 [0.71–0.96], *p* < 0.05), endocrine diseases (OR 0.67 [0.54–0.84], *p* < 0.05), injury, poisoning and other consequences of external causes (OR 0.60 [0.54–0.67], *p* < 0.05) and diseases of the musculoskeletal system and connective tissue (OR 0.69 [0.50–0.93], *p* < 0.05).

**Patient or Public Contribution:**

No patient or public contributions.

## 1 Introduction

In the field of public health nursing, epidemiology is used to assess the interaction of health determinants within the health‐illness continuum of both individuals and communities (Egry et al. 2018; Melo et al. 2021). Currently, the critical examination of social determinants by the nursing sector and the subsequent measures taken constitute the driving force behind the effort both to improve health outcomes for the population and to develop and consolidate our profession (Jones, Edwards, and Alexander 2022). One of the actions recommended by the Council of Public Health Nursing Organisations (CPHNO) is the operationalisation of health equity. In other words, it is necessary to identify and understand how social structures may affect clinical attendance, in order to enable the design of subsequent preventive interactions (Engle and Campbell 2019). Although the CPHNO presents this action in the framework of combating racism, public health nursing should also incorporate sex and gender as key health determinants, since they influence both the individual's self‐care and the way in which the health system responds to their needs (Malamou 2015).

In order to render the gender gap in health more visible, in 2019, for the first time, the World Health Organization (WHO) published its World Health Statistics disaggregated by sex, and called upon other institutions to follow its example, claiming that this would help health systems identify gender inequalities in health, understand how gender interacts with other factors to influence health outcomes and assign the necessary resources accordingly (Cabanillas‐Montferrer and Giménez‐Bonafé 2022). However, the gender differences present in the data have yet to be rendered completely visible in the field of health, and much the same can be said in terms of comparing health outcomes in accordance with sex (World Health Organization (WHO) 2023).

In‐hospital mortality is an important indicator for clinical and epidemiological research, often used to monitor the quality of care (García Ortega, Barrios, and García Ortega 1997). Many studies have analysed in‐hospital mortality by sex in specific pathologies such as acute myocardial infarction (Ribera et al. 2006; Rodríguez‐Padial et al. 2021; Roque et al. 2020), certain types of cancer (Abdel‐Fattah et al. 2022; Bruno et al. 2022; Lee et al. 2020; Sendra‐Gutiérrez et al. 2009; Taioli et al. 2017) and Alzheimer's disease (Golüke et al. 2019; Price et al. 2021; Shayne et al. 2013; Wang et al. 2014), among others. However, we failed to find any statistical databases that systematically compare differences in this indicator by sex or gender.

Such a comparison is necessary insofar as sex and gender clearly influence mortality: women have a greater life expectancy than men (Lopez‐de‐Andres et al. 2023), but suffer from poorer health for most of their lives (Darbà and Marsà 2021; Nakanishi, Yamasaki, and Nishida 2018). The difference in life expectancy may be explained by biological sex differences and the different behaviours that confirm gender, with neither one alone being wholly responsible (Carrillo‐Larco and Bernabé‐Ortiz 2018; Concepción‐Zavaleta et al. 2015; Nakanishi, Yamasaki, and Nishida 2018). From a physiological standpoint, the interaction between genetic differences, the role of sex hormones, the sexual dimorphism of the immune system and the distribution of body fat has an impact on morbidity and mortality (Carrillo‐Larco and Bernabé‐Ortiz 2018). For their part, gender differences linked to social functions and access to information, resources and preventive and curative measures also influence health and life expectancy (Moradabadi, Hannani, and Torkashvand 2023).

Overlooking the biological and social differences between men and women in terms of mortality may lead to biased clinical practice among nursing practitioners. In this line, Kuhn et al. (2017) found that emergency department nurses tended to allocate women with acute coronary syndrome a less priority triage category than men with the same symptoms, and that women waited longer for appropriate diagnostic tests, such as their first electrocardiograph. In addition, Prego‐Jimenez et al. (2022) reported, in a sample mainly comprised by registered nurses and physicians, that gender stereotypes could undermine the legitimation of low back pain, the willingness to offer support and credibility for female patients, but not for male patients. Therefore, it is crucial to consider both nurses' gender‐sensitive clinical judgement and patients' health literacy when assessing how health status or disease impacts life expectancy. These factors are vital components of nurse‐led gender‐sensitive health education. Indeed, research indicates that inadequate health literacy correlates with a heightened risk of mortality and hospitalisation (Franchi‐Alfaro et al. 2018; Salvador Marín et al. 2021), and studies have shown that women generally possess lower levels of health literacy compared to men (Kuhn et al. 2017).

In light of the above, it is vital for public health nursing to adopt a gender‐sensitive perspective when interpreting epidemiological results. However, we failed to find any statistical indicator that simultaneously screens for gender differences in all registered diseases of a population. Consequently, the aim of the present study is to analyse the association between gender and in‐hospital mortality odds ratios among patients from the Basque Country for each of the diagnostic categories included in the International Classification of Diseases 10 (ICD‐10) (Appendix I).

## 2 Methods

### 2.1 Design and Data Collection

A cross‐sectional retrospective study was carried out to analyse the association between gender, reason for hospital admission and in‐hospital mortality among patients in the Basque Country.

Admission data pertaining to the period between 1 January 2016 and 31 December 2018 were gathered for all registered acute care hospitals (both public and private) in the Basque Country. The data were provided by Eustat, the Basque Statistics Institute (Eustat 2020). The reason for admission was reflected through the principal diagnosis, which is established on the basis of the necessary examination and is identified as the cause of the patient's contact with the hospital (Ministerio de Sanidad Servicios Sociales e Igualdad 2015). Principal diagnoses were categorised in accordance with ICD‐10 codes (Appendix I).

### 2.2 Measures

#### 2.2.1 Independent Variable

*Gender* was categorised as man or woman on the basis of the information contained in the Set of Basic Minimum Data for Specialist Care in the Basque Country register.

#### 2.2.2 Dependent Variable

*Mortality* was categorised as a dichotomous variable: (a) discharged as deceased or (b) alive upon discharge. This latter group comprised patients who were discharged home, transferred to another hospital or social‐health centre or another possible destination.

#### 2.2.3 Control Variables

The variable *province* refers to the three provinces of the Basque Country: Araba, Gipuzkoa and Bizkaia. This variable indicates the location in which the patient received the corresponding medical attention.

*Age* was categorised in accordance with five groups: (a) ≤ 14 years, (b) 15–44 years, (c) 45–64 years, (d) 65–84 years and (e) ≥ 85 years.

Hospitals were categorised as either *public* or *private*, depending on the legal organisation or entity to which they belonged.

### 2.3 Statistical Analysis

In terms of descriptive statistics, frequencies and percentages were used to summarise the characteristics of our sample. Odds ratios (OR) were calculated through binomial logistic regressions to determine the association between gender and in‐hospital mortality for each diagnostic category. The assumptions of linearity, independence of errors and multicollinearity have been respected (Field 2013). We presented the corresponding OR and 95% confidence intervals adjusted by territory, hospital ownership and patient's age. Statistical significance was set at *p* < 0.05. The analyses were conducted using SPSS 29.0 (IBM Corp., Armonk, NY).

## 3 Results

As shown in Table 1, 45.6% of in‐hospital deaths in the Basque Country during the 3 years covered by the study correspond to women (as opposed to 54.4% corresponding to men). It should be noted that according to the total values for the 2016–2018 period, in both types of hospital (public and private), in all three provinces (Araba, Gipuzkoa and Bizkaia) and for all ages under 84 years, more men than women died in hospital. In contrast, among those aged ≥ 85 years, women had a higher in‐hospital mortality rate.

**TABLE 1:** Deaths in acute care hospitals of the Basque Country for the period 2016–2018.

	2016		2017		2018		Total 2016–2018	
	Women	Men	Women	Men	Women	Men	Women	Men
Total, *N* (%)^a^	3259 (2.80%)	3966 (3.44%)	3465 (3.00%)	4110 (3.51%)	3401 (2.93%)	4023 (3.37%)	10125 (2.91%)	12099 (3.44%)
Territory								
Araba	577 (3.23%)	675 (3.61%)	612 (3.45%)	715 (3.76%)	557 (3.06%)	679 (3.49%)	1746 (3.25%)	2069 (3.62%)
Gipuzkoa	1131 (2.87%)	1283 (3.30%)	1135 (2.96%)	1308 (3.40%)	1106 (2.92%)	1248 (3.23%)	3372 (2.92%)	3839 (3.31%)
Bizkaia	1551 (2.61%)	2008 (3.47%)	1718 (2.89%)	2087 (3.49%)	1738 (2.89%)	2096 (3.41%)	5007 (2.80%)	6191 (3.46%)
Hospital ownership								
Public	2867 (2.97%)	3608 (3.78%)	2927 (3.06%)	3539 (3.63%)	2849 (2.95%)	3414 (3.43%)	8643 (2.99%)	10561 (3.61%)
Private	392 (1.96%)	358 (1.80%)	538 (2.71%)	571 (2.91%)	552 (2.84%)	609 (3.07%)	1482 (2.50%)	1538 (2.59%)
Age								
≤ 14 years	16 (0.32%)	29 (0.44%)	13 (0.29%)	24 (0.37%)	33 (0.69%)	26 (0.40%)	62 (0.44%)	79 (0.40%)
15–44 years	64 (0.18%)	65 (0.38%)	44 (0.13%)	72 (0.42%)	52 (0.16%)	66 (0.39%)	160 (0.16%)	203 (0.40%)
45–64 years	386 (1.58%)	692 (2.08%)	409 (1.67%)	632 (1.88%)	398 (1.59%)	619 (1.80%)	1193 (1.61%)	1943 (1.92%)
65–84 years	1302 (3.54%)	2084 (4.37%)	1318 (3.59%)	2192 (4.49%)	1244 (3.39%)	2083 (4.18%)	3864 (3.51%)	6359 (4.35%)
≥ 85 years	1491 (9.61%)	1096 (10.30%)	1681 (10.11%)	1190 (10.48%)	1674 (9.81%)	1229 (10.24%)	4846 (9.85%)	3515 (10.34%)

^a^ Percentages of deceased women and men, according to the number of admitted women and men to acute care hospitals.

### 3.1 Risk of Death by Gender

**FIGURE 1:**
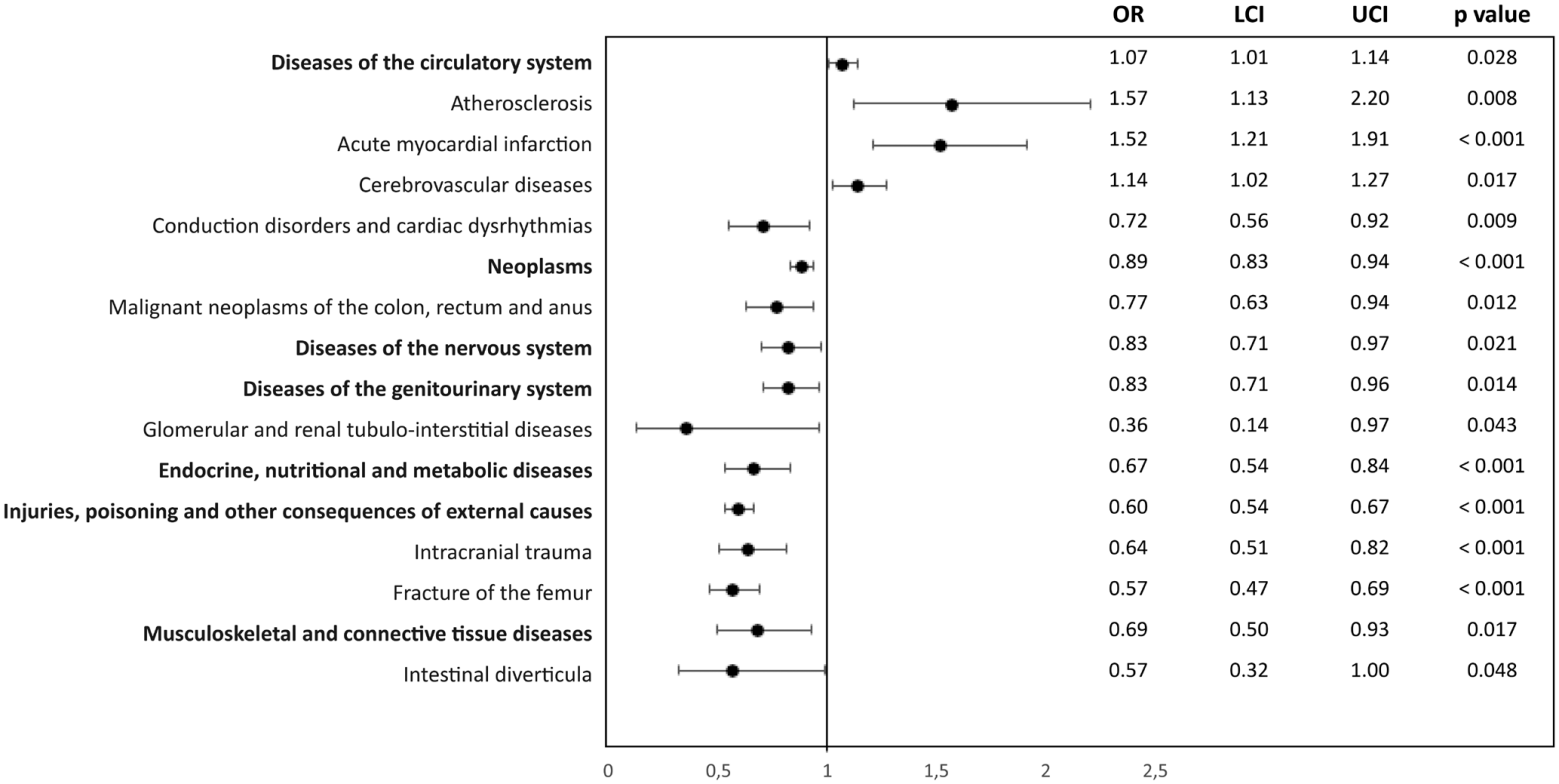
Adjusted odds ratio for in‐hospital mortality (men vs. women) by diagnosis. LCI, lower confidence interval; OR, odds ratio; UCI, upper confidence interval.

The coefficients obtained indicate that the odds ratio of dying in hospital were significantly higher for women than for men in relation to diseases of the circulatory system (OR 1.07 [1.01–1.14], *p* < 0.05), atherosclerosis (OR 1.57 [1.13–2.20], *p* < 0.05), acute myocardial infarction (OR 1.52 [1.21–1.91], *p* < 0.05) and cerebrovascular diseases (OR 1.14 [1.02–1.27], *p* < 0.05) (Figure 1).

For their part, men were more likely to die from neoplasms (OR 0.86 [0.83–0.94], *p* < 0.05), diseases of the nervous system (OR 0.83 [0.70–0.97], *p* < 0.05), diseases of the genitourinary system (OR 0.83 [0.71–0.96], *p* < 0.05), malignant neoplasms of the colon, rectum and anus (OR 0.77 [0.63–0.94], *p* < 0.05), cardiac conduction disorders and dysrhythmias (OR 0.72 [0.56–0.92], *p* < 0.05), diseases of the musculoskeletal system and connective tissue (OR 0.69 [0.50–0.93], *p* < 0.05), endocrine, nutritional and metabolic diseases (OR 0.67 [0.54–0.84], *p* < 0.05), intracranial trauma (OR 0.64 [0.50–0.82], *p* < 0.05), injury, poisoning and other consequences of external causes (OR 0.60 [0.54–0.67], *p* < 0.05), fracture of the femur (OR 0.57 [0.47–0.69], *p* < 0.05), intestinal diverticula (OR 0.57 [0.32–0.99], *p* < 0.05) and glomerular and tubulointerstitial diseases (OR 0.36 [0.14–0.97], *p* < 0.05) (Figure 1).

No statistically significant differences were found between genders in mortality for any of the other ICD‐10 diagnostic categories (Appendix II).

## 4 Discussion

The analysis of the association between gender and in‐hospital mortality for each ICD‐10 category for the 2016–2018 period in the Basque Country revealed several statistically significant differences. First, women were found to have a statistically higher in‐hospital mortality odds ratio than men in relation to diseases of the circulatory system. In contrast, for neoplasms, diseases of the nervous system, diseases of the genitourinary system, endocrine diseases, injury, poisoning and other consequences of external causes, diseases of the musculoskeletal system and connective tissue and intestinal diverticula, men were at a higher risk of death than women.

Consistently with the results found here, several other studies have also reported a higher risk of in‐hospital mortality among women diagnosed with acute myocardial infarction (Bruno et al. 2022; Rodríguez‐Padial et al. 2021; Roque et al. 2020), stroke (Abdel‐Fattah et al. 2022; Arboix et al. 2014) and atherosclerosis (Lee et al. 2020). Furthermore, despite the fact that only 30% of myocardial infarctions, including those with ST‐segment elevation, occur in women (Rodríguez‐Padial et al. 2021), acute myocardial infarction remains a leading cause of mortality among females (DeFilippis et al. 2020; Ibanez et al. 2018). This could be influenced by several factors, such as older age at the time of the event, a higher prevalence of atypical clinical presentation, a greater number of risk factors and differences in both the pathophysiology of the disease and the treatment received (Holtzman et al. 2023; Ibanez et al. 2018).

In relation to cerebrovascular diseases, these differences have been associated with women's greater life expectancy (since the incidence rate for stroke increases with age), as well as with a greater prevalence of this disease among females (Arboix et al. 2014).

As for neoplasms, the higher likelihood of in‐hospital mortality due to colorectal cancer observed among men by Pucciarelli et al. (2017) in Italy between 2005 and 2014 seems to be consistent with the higher odds ratio found among the men in our sample for death owing to malignant neoplasms of the colon, rectum and anus. In relation to lung cancer, no gender differences were observed in our sample. This is consistent with that reported by Taioli et al. (2017), who also failed to find differences after analysing a sample of patients with lung cancer who underwent limited resection or lobectomy between 1995 and 2012 in the state of New York. However, another study conducted in Spain in 2005 found a high rate of in‐hospital mortality among men admitted for the first time as a result of this type of cancer (Sendra‐Gutiérrez et al. 2009). The difference in the results found in our study and the one cited above may be due to the fact that the risk of in‐hospital mortality varies in accordance with time from diagnosis. A higher risk was found for men among newly‐admitted patients, whereas no gender differences were observed in our sample, which included both first time and recurrent admissions. Indeed, differences between men and women have been reported in relation to the course of lung cancer, since women receive more chemotherapy during their first hospital admission, have fewer adenocarcinomas and epidermoid tumours, smoke less and undergo fewer surgical procedures when readmitted than men (Sendra‐Gutiérrez et al. 2009). However, the differences between the samples in terms of inclusion criteria, location and year preclude any direct comparison of the results.

In the case of diseases of the nervous system, in the Basque Country, the likelihood of in‐hospital death was higher among men. In relation to this, some authors have observed a greater likelihood of in‐hospital death among men for the diagnostic subgroups dementia and/or Alzheimer's disease (Golüke et al. 2019; Lopez‐de‐Andres et al. 2023; Nakanishi, Yamasaki, and Nishida 2018). For example, in Japan, an observational study involving 960,423 people aged over 65 years who had died from Alzheimer's disease, vascular dementia or another kind of dementia, concluded that men were more at risk of dying in hospital than women (Nakanishi, Yamasaki, and Nishida 2018). In contrast, in Spain, from 2011 to 2016, the in‐hospital mortality rate was higher among female than among male Alzheimer's patients (Darbà and Marsà 2021). No statistically significant differences have been found between sexes for either epilepsy (Si et al. 2018) or multiple sclerosis (Pirttisalo et al. 2018). However, although some studies report a greater likelihood of in‐hospital death among men as a result of different types of dementia, a finding that is similar to that observed for diseases of the nervous system in our study, the results for other pathologies vary.

In relation to diseases of the genitourinary system, men in the Basque Country had a higher in‐hospital mortality rate than women. Consistently with this finding, in the United States, female patients with end‐stage renal disease on dialysis who are hospitalised with heart failure were found to be 25% less likely to die in hospital than their male counterparts (Inampudi et al. 2019). In contrast, Carrillo‐Larco and Bernabé‐Ortiz (2018) observed that the mortality rate for chronic renal disease was higher for women than for men (2.2% and 1.8% respectively); and Concepción‐Zavaleta et al. (2015) found no significant sex differences in relation to chronic end‐stage kidney disease. These inconsistencies between the results reported in the extant literature and those found here may be due to differences in the way diseases are categorised in the different studies. Furthermore, although in relation to glomerular and tubulointerstitial kidney diseases men in the Basque Country were found to be more likely to die in hospital than women, Beckwith, Lightstone, and McAdoo (2022) concluded that no statistically significant differences existed between men and women in terms of death from lupus nephritis and anti‐neutrophil cytoplasmic antibody‐associated vasculitis (two glomerular diseases). Nevertheless, some authors have attempted to explain the greater prevalence of these diseases among men in terms of a combination of biological, social, cultural and occupational factors. For example, women with the same level of creatinine as men have poorer kidney function due to the fact that they have less muscle mass. Also, exposure to hydrocarbons (a frequent occurrence in masculinised industrial sectors such as the painting profession and chemical industries), greater delays in seeking medical attention and more frequent smoking and drug abuse among men may explain the greater prevalence of these diseases among this sex (Beckwith, Lightstone, and McAdoo 2022).

Moving on to another ICD‐10 category, in relation to endocrine, nutritional and metabolic diseases, the in‐hospital mortality rate among men in our study was higher than that of women. In contrast, in a longitudinal retrospective study carried out in Ghana (Papadopoulos et al. 2008), the authors concluded that there were no significant differences between men and women in terms of in‐hospital mortality due to diseases of the endocrine system. In Iran, however, it was observed that from 2006 to 2018, more women than men had died from endocrine, nutritional and metabolic diseases (Moradabadi, Hannani, and Torkashvand 2023); although since the authors did not specify whether or not these deaths had occurred in hospital, it is difficult to directly compare these results with those found in our study.

For male patients in the Basque Country, the likelihood of dying in hospital from injury, poisoning or other consequences of external causes was higher than for women. Consistently with this finding, studies on severe trauma in men aged ≥ 60 years (Medina‐Molina, Balcells‐Martinez, and Prat‐Fabregat 2019) and vertebral fractures (Ong et al. 2018) report that the male population is at greater risk of in‐hospital death than the female one. In the diagnostic subgroup fracture of the femur, men in the Basque Country also had a greater likelihood than women of dying in hospital. Consistently with this finding, in a study carried out in Lombardy, the authors (Viganò et al. 2023) found a significantly higher risk of death among men one and 2 years after a hip fracture, and the same trend has been observed in the United States also, with the mortality rate among men with pelvic fractures being 10.2% higher than among women (Yoshihara and Yoneoka 2014). Nevertheless, other authors have failed to find any association between sex and likelihood of in‐hospital death for these same diagnoses (Salvador Marín et al. 2021) or have reported a greater risk among women (Franchi‐Alfaro et al. 2018). The variation between findings may be due to the specific particularities of each study in terms of how they interpret the ICD category injury, poisoning and other consequences of external causes in comparison with the general interpretation used here.

Finally, within this same ICD category, in relation to the specific diagnosis of intracranial trauma, in‐hospital mortality was higher among men in our study than among women. Consistently with this finding, in a study carried out in the USA between 2000 and 2017, the authors observed a statistically significant higher mortality rate among male than among female patients as a result of traumatic brain injury (Daugherty et al. 2019). In contrast, in Australia, although most in‐hospital deaths following traumatic brain injury corresponded to men (69.2%), no statistically significant differences were observed between men and women admitted as a result of this kind of injury (O'Reilly et al. 2023).

In the diseases of the musculoskeletal system and connective tissue category, men in the Basque Country were at greater risk of in‐hospital death than women. A similar trend was observed in a Spanish study on osteomyelitis, in which the authors observed that being a woman was a protective factor for in‐hospital mortality among patients suffering from this disease (López del Pino and Guerrero Espejo 2019). In Korea, although sex was not found to predict in‐hospital mortality among knee arthroplasty patients, it was found to predict postoperative mortality, with men being at greater risk than women (Choi et al. 2021).

Finally, for intestinal diverticula diagnoses, men from the Basque Country were at greater risk of in‐hospital death than women. In the United States also, women were found to have lower mortality rates in a study analysing a sample of 4 million hospital admissions for diverticulitis (Diamant et al. 2015). However, it is worth noting that, in Italy, in‐hospital mortality due to this pathology increased significantly for women between 2008 and 2015 (Binda et al. 2018).

If we focus on the general ICD categories, we see that, in our study, the odds ratios for in‐hospital death were higher for men suffering from neoplasms, diseases of the nervous system, diseases of the genitourinary system, injury and poisoning and diseases of the musculoskeletal system; whereas among women, the odds ratio for in‐hospital death was higher among those with diagnoses listed under the general diseases of the circulatory system category. Interestingly, we found no other study analysing these general ICD categories per se in accordance with gender. We are therefore unable to directly compare results, and have opted instead to discuss the risk of in‐hospital mortality in these general categories with the results reported in studies focusing on similar diseases. There is an absence in the extant literature of comparisons between men and women for many of the specific pathologies outlined in the ICD. This serves to highlight the lack of any systematic method for comparing a very basic indicator (namely in‐hospital mortality) in accordance with gender. Given the territorial characteristics of the Basque Country, one of the strengths of the present study is the homogeneity of the community sample analysed, as well as its large size. Furthermore, the fact that all data were obtained from the same source (Eustat—the Basque Statistics Institute) guarantees a high degree of standardisation in their processing. However, the analysis may have benefited from the study of more factors influencing health outcomes that, unfortunately, were not available, such as socioeconomic status, ethnic origin and reason for hospital admission.

### 4.1 Clinical Implications

Comparing in‐hospital mortality odds ratios by gender has been shown to be a good indicator for identifying those diseases in which the differences between men and women are greatest. It is now important to compare these results with those found in other populations. The analysis conducted here should therefore be replicated in other hospitals and statistical observatories, etc.

The present study highlights various biological specificities linked to gender that may influence mortality, including the pathogenesis of lung cancer and glomerular diseases. It is important for nursing practitioners to be aware of these physio‐pathological differences in order to avoid succumbing to type B gender bias, or in other words, assuming, when assessing a patient, that the physio‐pathologies of men and women are the same when, in fact, they are quite different (Cabanillas‐Montferrer and Giménez‐Bonafé 2022).

Following Henderson's model (Correa Argueta, Verde Flota, and Rivas Espinosa 2016), several sources of difficulties have been detected in the vulnerable population that could be modified in order to enable people to reach their full health potential. For example, the lower awareness of the importance of acute myocardial infarction among women may be redressed through primary care nurse‐led health education. In this sense, it is worth noting that a nurse‐led phone follow‐up education programme proved effective in increasing self‐efficacy for disease management in a study involving 403 patients of both sexes suffering from cardiovascular disease (Zhou et al. 2018). Similarly, nurse‐led intervention initiatives such as the Protecting Healthy Hearts Program may be useful for improving the management of cardiovascular risk factors such as total cholesterol, weight and blood pressure (Carrington and Stewart 2015). These initiatives were based on individual plans for developing self‐care skills, promoting healthy lifestyles and therapeutic adherence with follow‐ups scheduled in accordance with each patient's risks and needs. They managed to reduce systolic blood pressure by 4 mmHg, diastolic blood pressure by 1 mmHg and body mass index by 0.3 kg/m^2^, among others (Carrington and Stewart 2015).

Delays in seeking medical assistance among women constitute another factor linked to their higher in‐hospital mortality rate due to coronary disease (Holtzman et al. 2023). This same factor is also associated with higher in‐hospital mortality rates among men due to glomerular diseases (Beckwith, Lightstone, and McAdoo 2022). Promoting health at all ages and levels is the best possible tool for reducing the time that elapses before patients seek medical attention (Moreno‐Martínez et al. 2016). This can be achieved through interventions targeting women's lifestyle and the principal cardiovascular risk factors, as well as through preventive pharmacological interventions. In all these actions, the active participation of the nursing profession is vital (Wood and Gordon 2012). These same interventions could be adapted for glomerular diseases.

Smoking has been linked to greater in‐hospital mortality due to lung cancer (Sendra‐Gutiérrez et al. 2009) and glomerular diseases (Beckwith, Lightstone, and McAdoo 2022) among men. Nursing has a key role to play in both preventing smoking and helping people to give up once they have started. For example, an intensive nurse‐led intervention programme targeted at 163 patients, carried out from 2004 to 2012 at a health centre in Asturias, achieved a smoking abstinence rate of 45.1% 12 months later (Blanco Riopedre and Fernández Fernández 2015).

Finally, occupational exposure to hydrocarbons may be a risk factor for lupus nephritis and anti‐neutrophil cytoplasmic antibody‐associated vasculitis (Beckwith, Lightstone, and McAdoo 2022). In relation to this, it is important to highlight the role of occupational nursing, not only in the protection, prevention and promotion of health in the labour field, but also in terms of monitoring workers' health, working conditions and the risks present in the workplace (Juárez‐García and Hernández‐Mendoza 2010).

Across its entire scope of action and from the perspective of public health and occupational health, nursing therefore has the capacity to intervene to prevent those factors that may contribute to higher rates of in‐hospital mortality.

### 4.2 Study Limitations

Our study has certain limitations that should be considered when interpreting the findings. One of the main limitations of this study is the nature of the data set, which does not allow us to identify deceased patients or to obtain details on their comorbidities or medical history. The lack prevents an exhaustive analysis of the factors that could have influenced the prognosis and evolution of the patients, which could limit the generalisation of the results. In addition, the subjectivity and variability that may exist among clinicians when assigning an ICD‐10 diagnostic code to individuals upon admission or death must be considered. Finally, we recognise that the results obtained in this study should be interpreted with caution, as they include only in‐hospital mortality, and not out‐of‐hospital mortality. Despite these limitations, we believe that the results obtained provide valuable information on gender inequalities in in‐hospital mortality and provide a solid basis for future research.

## 5 Conclusions

Whereas in relation to diseases of the circulatory system women were more likely to die in hospital, for all the other diseases analysed, men had higher in‐hospital mortality rates.

To the best of our knowledge, this is the first study to analyse the odds ratios of in‐hospital mortality by gender for each of the ICD‐10 categories.

Having this overview of our health system will enable us to determine a starting point for working towards equality between men and women within the health service.

There are many different factors (biology, gender roles in society, life expectancy, literacy, health actions, etc.) that may explain these inequalities. Being aware of the ICD‐10 categories in which these inequalities exist may help us better organise our resources in order to continue researching this issue, since remaining unaware of the factors that may be causing these differences does nothing but perpetuate a situation of inequality between men and women.

Viewing prevention as the cornerstone of the public health system, and nursing practitioners as key professionals within that system, we hope this paper has served to highlight the importance of working, from within the nursing sector, on health promotion activities, with the aim of influencing those factors that may result in men and women having unequal health outcomes.

## Author Contributions

All of the authors contributed intellectually to the work, meet the conditions of authorship and have approved the final version of it. A.L. devised the project, the main conceptual ideas and proof outline, N.Z.‐Z., U.E.‐G. and H.A. analysed the data, B.P.‐G. and N.V.‐A. developed the theoretical framework and N.V.‐A., M.U.‐Y. and J.Z.‐E. discussed the results. I declare that the work is original, has not been previously published and is not under review by any other journal. Ethical principles have been consistently adhered to throughout the research process, and we would be willing to provide more information about our data and methods if necessary.

## Ethics Statement

In compliance with the Spanish Biomedical Research Law (14/2007), this study was exempt from Institutional Review Board approval as it solely encompasses the analysis of publicly available non‐nominal data.

## Conflicts of Interest

The authors declare no conflicts of interest.

## Data Availability

The data supporting the findings of this study are available at Eustat—the Basque Institute of Statistics.

## Appendix I International Classification of Diseases (ICD‐10)

- (A00–B99) Certain infectious and parasitic diseases
- (C00–D49) Neoplasms
- (D50–D89) Diseases of the blood and blood‐forming organs and certain disorders involving the immune mechanism
- (E00–E89) Endocrine, nutritional and metabolic diseases
- (F01–F99) Mental and behavioural disorders
- (G00–G99) Diseases of the nervous system
- (H00–H59) Diseases of the eye and adnexa
- (H60–H95) Diseases of the ear and mastoid process
- (I00–I99) Diseases of the circulatory system
- (J00–J99) Diseases of the respiratory system
- (K00–K95) Diseases of the digestive system
- (L00–L99) Diseases of the skin and subcutaneous tissue
- (M00–M99) Diseases of the musculoskeletal system and connective tissue
- (N00–N99) Diseases of the genitourinary system
- (O00–O9A) Pregnancy, childbirth and the puerperium
- (P00–P96) Certain conditions originating in the perinatal period
- (Q0–Q99) Congenital malformations, deformations and chromosomal abnormalities
- (R00–R99) Symptoms, signs and abnormal clinical and laboratory findings, not elsewhere classified
- (S00–T88) Injury, poisoning and certain other consequences of external causes
- (V00–Y99) External causes of morbidity and mortality
- (Z00–Z99) Factors influencing health status and contact with health services
- (U00–U85) Codes for special purposes

## Appendix II Adjusted Analysis for In‐Hospital Mortality (Women vs. Men) for Each Diagnostic Category

**Table:**

International Classification of Diseases 10 (ICD‐10)	*p*	OR	CI 95%	
			Lower	Upper
**Certain infectious and parasitic diseases**	0.448	0.96	0.87	1.07
Intestinal infectious diseases, except diarrhoea	0.906	1.05	0.50	2.20
Infectious gastroenteritis and colitis	0.677	1.36	0.32	5.84
Tuberculosis	0.444	0.58	0.14	2.35
Septicaemia	0.464	1.04	0.93	1.17
Human immunodeficiency virus [HIV] disease	0.886	0.94	0.40	2.19
Other infectious and parasitic diseases	0.973	0.99	0.69	1.44
**Neoplasms**	< 0.001	0.89	0.83	0.94
Malignant neoplasms of the colon, rectum and anus	0.012	0.77	0.63	0.94
Malignant neoplasms of the trachea, bronchus and lung	0.863	0.98	0.81	1.19
Melanoma and other malignant skin neoplasms	0.394	0.74	0.37	1.48
Malignant neoplasm of the breast	0.972	1.04	0.14	7.73
Malignant neoplasm of the bladder	0.719	1.08	0.71	1.66
Other malignant neoplasms	0.472	1.03	0.95	1.12
Carcinoma in situ	0.600	0.62	0.10	3.77
Benign neoplasm of the colon, rectum, anal canal and anus	0.464	0.42	0.04	4.23
Other benign neoplasms and neoplasms of uncertain or unknown behaviour or unknown	0.019	0.64	0.44	0.93
**Diseases of the blood and blood‐forming organs and certain disorders involving the immune mechanism**	0.994	1.00	0.74	1.36
Anaemias	0.922	1.02	0.68	1.53
Other diseases of the blood and haematopoietic organs and disorders affecting the immune mechanism	0.482	1.18	0.74	1.88
**Endocrine, nutritional and metabolic diseases**	< 0.001	0.67	0.54	0.84
Diabetes mellitus	0.734	1.07	0.71	1.61
All other endocrine, nutritional and metabolic diseases	< 0.001	0.47	0.36	0.61
**Mental and behavioural disorders**	0.054	0.61	0.37	1.01
Dementia	0.920	1.05	0.43	2.52
Mental and behavioural disorders due to alcohol use	0.541	0.51	0.06	4.34
Schizophrenia, schizotypal and delusional disorders	0.908	0.84	0.05	14.89
Mood (affective) disorders	0.693	0.70	0.11	4.21
Other mental and behavioural disorders	0.053	0.44	0.19	1.01
**Diseases of the nervous system**	0.021	0.83	0.71	0.97
Alzheimer's disease	0.694	0.84	0.36	1.99
Epilepsy	0.678	1.10	0.71	1.69
Transient cerebral ischaemia	0.532	0.61	0.13	2.83
Rest of (G00–G99) Other nervous system diseases	0.012	0.79	0.66	0.95
**Diseases of the ear and mastoid process**	0.340	0.33	0.03	3.21
**Diseases of the circulatory system**	0.028	1.07	1.01	1.14
Hypertensive disease	0.147	0.83	0.65	1.07
Angina pectoris	0.125	1.77	0.85	3.67
Acute myocardial infarction	< 0.001	1.52	1.21	1.91
Other ischaemic heart diseases	0.269	1.32	0.81	2.15
Pulmonary heart disease and diseases of the pulmonary circulation	0.575	1.10	0.79	1.54
Cardiac conduction disorders and dysrhythmias	0.009	0.72	0.56	0.92
Heart failure	0.340	0.94	0.83	1.06
Cerebrovascular diseases	0.017	1.14	1.02	1.27
Atherosclerosis	0.008	1.57	1.13	2.20
Varicose veins of the lower extremities	0.466	2.29	0.25	21.24
Other diseases of the circulatory system	0.002	0.75	0.62	0.90
**Diseases of the respiratory system**	0.199	0.96	0.90	1.02
Acute upper respiratory infections and influenza	0.434	1.13	0.83	1.54
Pneumonia	0.397	0.94	0.81	1.09
Other acute lower respiratory tract infections	0.001	0.66	0.52	0.84
Other upper respiratory tract diseases	0.676	0.62	0.06	5.95
Chronic obstructive pulmonary disease and bronchiectasis	0.151	0.84	0.67	1.06
Asthma	0.264	1.72	0.66	4.48
Other respiratory tract diseases	0.184	0.94	0.86	1.03
**Diseases of the digestive system**	0.182	0.94	0.86	1.03
Other diseases of the oral cavity, salivary glands and jaws	0.820	1.12	0.41	3.09
Diseases of the oesophagus	0.285	0.60	0.24	1.52
Gastric, duodenal and peptic ulcers	0.512	1.22	0.67	2.21
Dyspepsia and other diseases of the stomach and duodenum	0.104	0.54	0.25	1.14
Diseases of the Appendix	0.213	0.50	0.17	1.48
Inguinal hernia	0.204	1.78	0.73	4.34
Other abdominal hernia	0.151	0.70	0.43	1.14
Crohn's disease and ulcerative colitis	0.249	0.51	0.16	1.61
Other gastroenteritis, non‐infectious colitis and unspecified colitis	0.665	0.91	0.58	1.41
Paralytic ileus and intestinal obstruction without hernia	0.591	1.06	0.85	1.34
Intestinal diverticula	0.048	0.57	0.32	1.00
Diseases of the anus and rectum	0.405	1.28	0.72	2.28
Other diseases of the intestine	0.033	0.76	0.60	0.98
Alcoholic liver disease	0.777	0.92	0.54	1.59
Other liver diseases	0.675	0.94	0.69	1.27
Cholelithiasis	0.494	0.86	0.57	1.31
Other diseases of the gallbladder and biliary tract	0.961	0.99	0.71	1.39
Pancreatic diseases	0.821	0.96	0.64	1.42
Other diseases of the digestive tract	0.092	0.82	0.64	1.03
**Diseases of the skin and subcutaneous tissue**	0.735	0.94	0.64	1.37
Infections of the skin and subcutaneous tissue	0.550	0.82	0.43	1.57
Other skin and subcutaneous tissue diseases	0.779	0.93	0.58	1.51
**Diseases of the musculoskeletal system and connective tissue**	0.017	0.69	0.50	0.93
Coxarthrosis	0.556	0.59	0.10	3.46
Gonarthrosis	0.870	1.22	0.11	13.56
Other arthropathies	0.570	1.31	0.51	3.34
Systemic connective tissue disorders	0.104	3.54	0.77	16.29
Deforming dorsopathies and spondylopathies	0.072	0.42	0.17	1.08
Intervertebral disc disorder	0.185	0.23	0.03	2.01
Dorsalgia	0.552	0.65	0.16	2.68
Soft tissue disorders	0.501	0.81	0.43	1.51
Other musculoskeletal and connective tissue diseases	0.005	0.43	0.24	0.77
**Diseases of the genitourinary system**	0.014	0.83	0.71	0.96
Glomerular and renal tubulointerstitial diseases	0.043	0.36	0.14	0.97
Renal insufficiency	0.242	0.87	0.70	1.09
Urinary lithiasis	0.394	0.50	0.10	2.48
Other diseases of the urinary system	0.939	0.99	0.78	1.26
Other disorders of the genitourinary system	< 0.001	0.11	0.03	0.37
**Certain conditions originating in the perinatal period**	0.957	0.99	0.61	1.60
Disorders related to short gestation and low birth weight, not elsewhere classified	0.776	1.10	0.56	2.17
Other conditions originating in the perinatal period	0.602	0.83	0.41	1.67
**Congenital malformations, deformations and chromosomal abnormalities**	0.154	1.69	0.82	3.47
**Symptoms, signs and abnormal clinical and laboratory findings, not elsewhere classified**	0.696	0.97	0.83	1.13
Throat and chest pain	0.132	0.42	0.13	1.30
Abdominal pain	0.721	1.14	0.55	2.37
All other symptoms, signs and abnormal clinical and laboratory findings, not elsewhere classified	0.632	1.04	0.88	1.23
**Injury, poisoning and certain other consequences of external causes**	< 0.001	0.60	0.54	0.67
Intracranial trauma	< 0.001	0.64	0.51	0.82
Other head injuries	0.811	1.08	0.57	2.04
Fracture of the femur	< 0.001	0.57	0.47	0.69
Fracture of the leg, including the ankle	0.063	0.26	0.06	1.07
Other trauma	0.004	0.62	0.45	0.86
Burns and corrosions	0.478	0.56	0.11	2.80
Poisonings from drugs, medicines and biological substances and toxic effects of non‐medicinal substances	0.544	0.75	0.30	1.88
Complications of surgical and medical care, not elsewhere classified other	0.229	0.85	0.65	1.11
Other effects and unspecified effects of external causes	0.712	0.94	0.69	1.28
**Factors influencing health status and contact with health services**	< 0.001	0.35	0.29	0.43
Observation and evaluation for suspected illnesses not found	< 0.001	0.20	0.08	0.46
Other medical care (including radiotherapy and chemotherapy sessions)	0.258	0.85	0.65	1.12
All other factors influencing health status and contact with health services	0.001	0.45	0.28	0.74

*Note:* Bold text indicates general ICD categories.
